# Lb1G04202, an Uncharacterized Protein from Recretohalophyte *Limonium bicolor*, Is Important in Salt Tolerance

**DOI:** 10.3390/ijms23105401

**Published:** 2022-05-12

**Authors:** Xi Wang, Baoshan Wang, Fang Yuan

**Affiliations:** Shandong Provincial Key Laboratory of Plant Stress, College of Life Sciences, Shandong Normal University, Ji’nan 250014, China; cici934384418@163.com

**Keywords:** *Limonium bicolor*, heterologous expression, osmotic stress, salt resistance, salt gland

## Abstract

With global increases in saline soil, it has become increasingly important to decipher salt-tolerance mechanisms and identify strategies to improve salt tolerance in crops. Halophytes complete their life cycles in environments containing ≥200 mM NaCl; these remarkable plants provide a potential source of genes for improving crop salt tolerance. Recretohalophytes such as *Limonium bicolor* have salt glands that secrete Na^+^ on their leaf epidermis. Here, we identified *Lb1G04202*, an uncharacterized gene with no conserved domains, from *L. bicolor*, which was highly expressed after NaCl treatment. We confirmed its expression in the salt gland by in situ hybridization, and then heterologously expressed *Lb1G04202* in *Arabidopsis thaliana*. The transgenic lines had a higher germination rate, greater cotyledon growth percentage, and longer roots than the wild type (WT) under NaCl treatments (50, 100 and 150 mM). At the seedling stage, the transgenic lines grew better than the WT and had lower Na^+^ and malonyldialdehyde accumulation, and higher K^+^ and proline contents. This corresponded with the high expression of the key proline biosynthesis genes *AtP5CS1* and *AtP5CS2* under NaCl treatment. Isotonic mannitol treatment showed that *Lb1G04202* overexpression significantly relieved osmotic stress. Therefore, this novel gene provides a potential target for improving salt tolerance.

## 1. Introduction

Irrigated soils are becoming increasingly salinized worldwide [1]. According to the Food and Agriculture Organization Harmonized World Soil Database, soil salinization affects one-third of river basins to some extent [2]. In 2021, more than 20% of global irrigated farmland was affected by soil salinization [3]. It is estimated that 12 million hectares of irrigated agricultural land may no longer be usable in 2050 due to soil salinization [4]. Salinization decreases the area of cultivated land, which seriously affects food production and food security [5,6,7]. Therefore, it is extremely urgent to develop, utilize, and improve saline land [8]. However, physical methods to improve saline soil are costly and unsustainable; therefore, it is particularly important to use biological methods to improve saline soil [9].

Most plants are sensitive to salinity, but some plants, called halophytes, grow normally and complete their life cycles in naturally occurring saline soil (≥200 mM NaCl), and there are three different types of halophytes according to the various salt resistance mechanisms [6]. Pseudohalophytes (salt excluders), such as *Phragmites australis*, prevent salt from entering the root stele with an apoplast barrier in the roots to maintain low levels of salt in the aboveground [6,10]. Euhalophytes, such as *Suaeda salsa*, are typical halophytes that compartmentalize salt in the central vacuole to avoid damage to other organelles [6,11]. Finally, recretohalophytes excrete salt from unique epidermal structures at organ level such as salt glands (such as *Limonium bicolor* [12]) or salt bladders (such as *Chenopodium quinoa* [13]). The salt secretory structures can help to secrete excess Na^+^ out of the plants to avoid salt damage. These specific structures or regulation mechanisms enable halophytes to grow normally on saline land and complete their life cycle [8]. 

Generally, halophytes develop common mechanisms that increase salt tolerance, such as increased cellular organic osmolyte contents and ion compartmentation for osmotic adjustment. Under salt stress, plants decompose starch to produce soluble sugar, increase cell fluid concentration and reduce osmotic potential [14]. Halophytes can also increase proline and K^+^ content (some halophytes can accumulate amounts of Na^+^ instead of K^+^) to reduce osmotic potential and prevent plant water loss [15]. In addition to these characteristics, recretohalphytes have evolved specific salt secretory epidermal structures to secrete excess salt out of the plant.

The roles of salt glands in salt resistance are of particular interest due to their presence in the leaf epidermis [16]. Unlike other halophytes, salt glands endow recretohalophytes with particular morphological and physiological mechanisms to resist salt damage [17]. Excess Na^+^ can be directly secreted from plants via the secretory pores of the salt glands. NaCl crystals form on the leaf surface of *L. bicolor* [18] and *Distichlis spicata* [19]. Three hypotheses have been proposed to explain the salt secretion mechanism: the osmotic hypothesis [20], the animal fluid transport similarity hypothesis [21], and vesicle-mediated exocytosis [22,23]. There is increasing evidence that vesicle-mediated transport plays an essential role in salt secretion, in which vesicles collect the excess ions and secrete them out of the cells [24,25,26].

*Limonium bicolor* could play an important role in improving saline environments by secreting excess salt from glands on stems and leaves [27,28]. It has a potential role for practical use in environment restoration through desalinization in practice. Additionally, it can also be used as a model halophyte to study salt resistance mechanisms related to salt gland development and salt secretion. The salt glands of *L. bicolor* have four spontaneous blue fluorescent foci, which can be clearly seen under a fluorescence microscope [29]. Moreover, a stable transformation system [29] and a virus-induced gene silencing (VIGS) system [26] are available for functional validation at the molecular level. Therefore, it is possible to identify genes involved in salt gland development and salt resistance in *L. bicolor*. Using the leaf development transcriptome of *L. bicolor*, many genes have been proposed to participate in salt resistance, including *LbTTG1* (*TRANSPARENT TESTA GLABRA1*), *LbSAD2* (*SUPER SENSITIVE TO ABA AND DROUGHT2*) and *LbTRY* (*TRIPTYCHON*) [30,31,32]. In addition, on the basis of the analysis of transcriptome data, we found many genes highly expressed during the development of salt glands; however, these genes have very low or no sequence identity with published plant genome sequences, such as *LbHLH*, which promotes salt resistance [33].

At present, most studies on the regulation of plant development and growth report known genes or proteins. For example, *TLR1* (*TrichomeLess Regulator1*) and *TLR2* (MYB transcription factor) negatively regulate trichome density and artemisinin levels in *Artemisia annua* [34]. Strigolactone (SLS) can regulate plant branching and other development processes [35], as can other genes in this species [30,31,32]. However, genes with undefined functions could also play important roles in plant vegetative growth, developmental regulation, and reproductive metabolism [33,36,37,38,39]. For example, in Arabidopsis, the newly identified protein IbSUT4 (sucrose transporters4) from *Ipomoea batatas* regulates the expression of *AtFT* (*FLOWERING LOCUS T*) and promotes growth, prolonging flowering time of plants. The promoter region of *IbSUT4* contains an ABRE motif, which induces the expression of ABA signaling pathway genes and inhibits the expression of *ABI1* (*ABA insensitive1*) [38].

By analyzing the leaf developmental transcriptome [40] and NaCl-treated salt secretion transcriptome [18] of *L. bicolor*, we found that several uncharacterized genes were highly expressed in early leaf development and under high-NaCl conditions. Given that no reference genome has been reported in species with salt glands, these novel genes with unknown functions have been proposed to participate in salt gland development or salt resistance in recretohalophytes. Among these unknown candidate genes, *Lb1G04202* showed high expression under NaCl treatment and was chosen for further analysis. Therefore, heterologous expression was carried out in Arabidopsis to investigate the potential role of the novel gene *Lb1G04202* from *L. bicolor*. Given that *Lb1G04202* was selected based on salt gland development, it is expected that this gene may play an important role in salt tolerance. The possible mechanism of enhancement in salt tolerance was also discussed, which will be further applied in saline transformation in the future. 

## 2. Results

### 2.1. Lb1G04202 Encodes an Uncharacterized Protein, Is Expressed in Salt Glands and Is Highly Expressed after Salt Treatment

**Figure 1:**
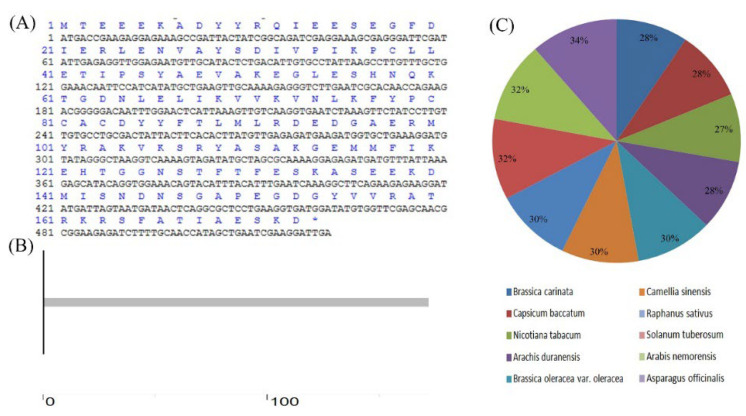
Bioinformatic analysis of *Lb1G04202.* (**A**) Protein translated by *Lb1G04202*. *Lb1G04202* contains 522 base pairs and encodes 173 amino acids. (**B**) Structural prediction of Lb1G04202. Lb1G04202 has no conserved domain or low complexity region. (**C**) Homology analysis between *Lb1G04202* of *Limonium bicolor* and other species. The genetic similarity between *Lb1G04202* of *L. bicolor* and other species was no more than 35%.

The full-length coding sequence of *Lb1G04202* from *L. bicolor*. *Lb1G04202* contains 522 base pairs and encodes 173 amino acids was cloned (Figure 1A). Lb1G04202 has no conserved domain or low-complexity region (Figure 1B). There were no genes from other species with more than 35% similarity with *Lb1G04202* (Figure 1C) and the genes with limited similarity were all uncharacterized or hypothetical. This indicates that *Lb1G04202* from *L. bicolor* is a unique and novel gene.

**Figure 2:**
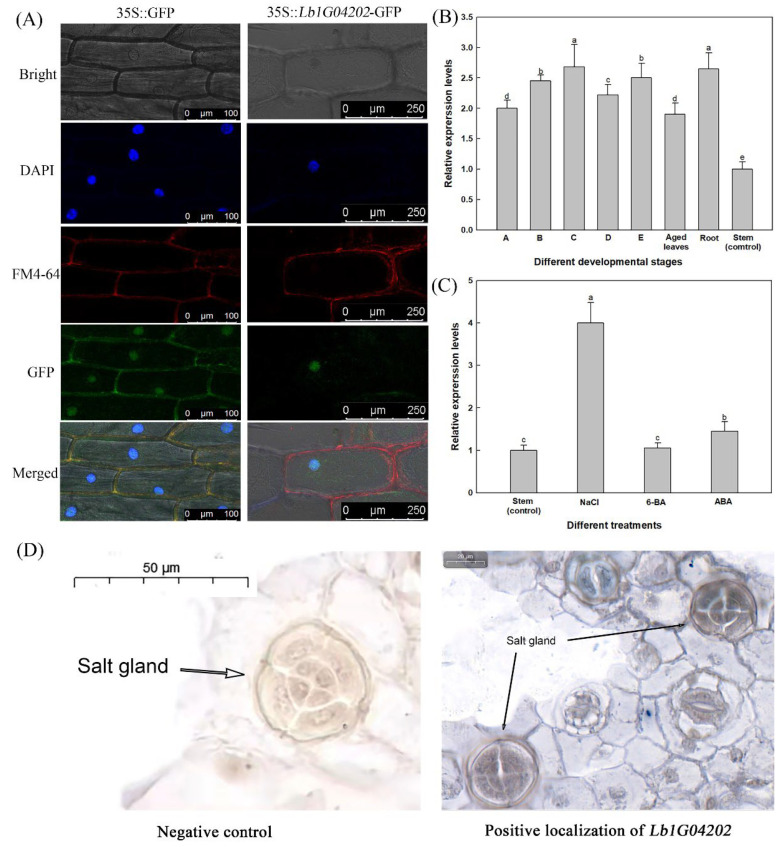
Localization and expression level analysis of *Lb1G04202*. (**A**) Subcellular localization analysis of 35S::*Lb1G04202*-GFP in onion epidermal cells. The GFP-Lb1G04202 fusion protein was expressed in the nucleus only. 35S::GFP was used as the empty vector control. Bar = 50 µm. DAPI appears as blue fluorescence specifically in the nucleus and FM4-64 appears as red fluorescence at the plasma membrane. (**B**) *Lb1G04202* expression levels in *Limonium bicolor* at developmental stages (A–E) and in different tissue parts. A: undifferentiated stage, 4–5 d after sowing; B: salt gland differentiation stage, 6–7 d after sowing; C: stomatal differentiation stage, 8–10 d after sowing; D: epidermal differentiation stage, 11–16 d after sowing; E: mature stage, more than 17 d after sowing; Aged leaves: fully expanded leaves. Roots and stems were collected from mature plants. Data are the means ± SD of three replicates. Different letters indicate significant differences at *p* = 0.05 according to Duncan’s multiple range test. (**C**) *Lb1G04202* expression levels in *L. bicolor* under different treatments. NaCl: mature leaves from stage E seedlings after 24 h of 200 mM NaCl treatment; 6-BA: mature leaves from stage E seedlings grown on 0.04 mg/L 6-BA; ABA: mature leaves from stage E seedlings grown on 0.1 mg/L ABA. (**D**) In situ hybridization of *Lb1G04202* using stage B–D leaves from *L. bicolor*. Negative control: the probe did not connect any nucleic acid sequence and could not detect any transcripts. Positive localization of *Lb1G04202*: *Lb1G04202* transcripts were detected using a digoxin-labeled anti-sense probe, which produces a blue-violet color.

Subcellular localization analysis and in situ hybridization for *Lb1G04202* in *L. bicolor* was conducted (Figure 2). DAPI (stains the nucleus) and FM4-64 (stains the membrane) were applied to determine the subcellular localization of *Lb1G04202* after transformation with the p35S::*Lb1G04202*-GFP expression vector in onion epidermal cells. As shown in Figure 2A, *Lb1G04202*-GFP was only located in the nucleus, while GFP was located in both the nucleus and plasma membrane. 

Furthermore, the expression position of *Lb1G04202* was determined using in situ hybridization to confirm its relationship with salt glands. As shown in Figure 2D, the hybridization signal was detected in the salt gland. We next determined the expression pattern of *Lb1G04202* in different plant tissue at various developmental stages (Figure 2B) and under different salt treatments (100 mM NaCl, 0.04 mg/L 6-BA and 0.1 mg/L ABA; Figure 2C). *Lb1G04202* expression was highest (relative to stem tissue as the control) under 100 mM NaCl treatment (Figure 2C), indicating that *Lb1G04202* is related to salt stress.

### 2.2. Heterologous Expression of Lb1G04202 Increases Arabidopsis Salt Resistance during Germination

**Figure 3:**
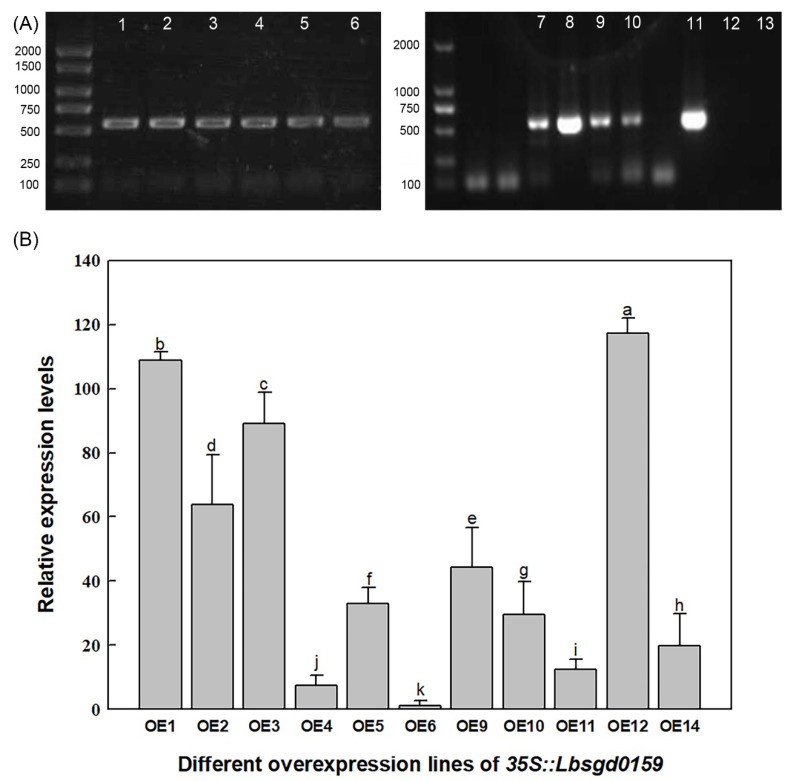
Molecular identification and expression analysis of Arabidopsis Col-35S::*Lb1G04202* lines. (**A**) PCR of genomic DNA from Col-35S::*Lb1G04202* lines. Lanes 1–6, 7–10 and 11, different transgenic lines; lane 12, blank control with ddH_2_O used as a template; lane 13, negative control with wild-type DNA used as a template. (**B**) Expression levels of *Lb1G04202* in Col-35S::*Lb1G04202* lines examined by qRT-PCR. Data are means ± SD of three replicates; different letters indicate significant differences at *p* = 0.05 according to Duncan’s multiple range test.

After transformation of Arabidopsis using the pCAMBIA3301 vector, 14 lines were obtained by Basta selection. Eleven positive Col-35S::*Lb1G04202* lines were identified at the DNA level (Figure 3A). After analyzing the expression level of *Lb1G04202* in the 11 transgenic lines (Figure 3B), OE1, OE2, and OE11 were selected as high, medium, and low expression level lines of Col-35S::*Lb1G04202*, respectively, for further experiments.

**Figure 4:**
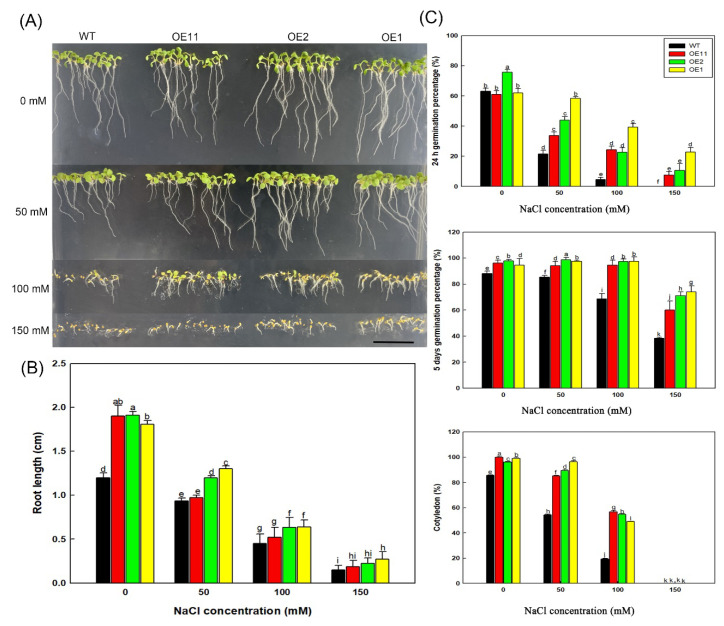
*Lb1G04202* increased the salt resistance of Arabidopsis during germination. (**A**) Col-35S::*Lb1G04202* seeds grown on medium with different concentrations of NaCl (0, 50, 100, and 150 mM) for 5 d. Bar = 1 cm. (**B**) Root lengths of 5-d-old seedlings were determined using ImageJ software. Thirty seedlings were analyzed per line. Root length data are the mean ± SD of 30 plants. (**C**) Analysis of germination levels and cotyledon growth under different NaCl treatments. Germination percentage was measured at 48 h and 5 d after sowing. Forty seeds per line were sown per treatment, and three replicates were performed. Germination percentage data are the mean ± SD of 40 plants. The cotyledon growth rate (expressed as the percentage of plants with emerged cotyledons) was calculated 3 d after sowing on different media. Forty seeds per line were sown per treatment, and three replicates were performed. Cotyledon growth rate data are mean ± SD of 40 plants. Different letters indicate significant differences at *p* = 0.05 according to Duncan’s multiple range test.

We investigated the effect of *Lb1G04202* on salt tolerance in Arabidopsis grown in three NaCl concentrations (50, 100 and 150 mM) and a control (0 mM). As shown in Figure 4A, the transgenic lines, especially OE1 and OE2, had higher growth under the NaCl treatments than the WT. The three Col-35S::*Lb1G04202* lines had a higher germination rate compared with the WT (Figure 4C) at both 24 h and 5 d after sowing under the 50, 100, and 150 mM NaCl treatments, particularly under the 150 mM NaCl treatment. None of the WT seeds had germinated by 24 h under the 150 mM NaCl treatment. There were minimal differences in germination rate among the different lines under the 0, 50, and 100 mM NaCl treatments. However, under the 150 mM NaCl treatment, the germination rates of the three Col-35S::*Lb1G04202* lines were significantly (almost two-fold) higher than that of the WT.

The differences among lines in cotyledon growth percentage were more obvious than the differences in germination rate (Figure 4C). In the absence of NaCl, the cotyledon growth rate was similar among the four lines. However, under the 50 and 100 mM NaCl treatments, the cotyledon growth of Col-35S::*Lb1G04202* lines was much higher than that of the WT. Under the 150 mM NaCl treatment, none of the four lines had cotyledons. Root growth was inhibited by NaCl. However, under each NaCl treatment, the root length of Col-35S::*Lb1G04202* lines was longer than that of the WT (Figure 4B).

### 2.3. Lb1G04202 Reduces NaCl Damage at the Seedling Stage

**Figure 5:**
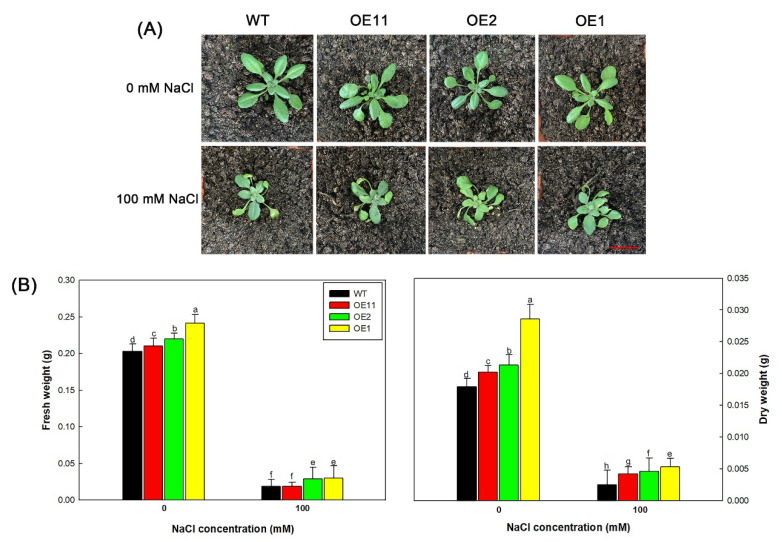
*Lb1G04202* increased the salt resistance of Arabidopsis at the seeding stage. (**A**) Growth of transgenic seedlings treated with Hoagland solution containing different concentrations of NaCl (0 and 100 mM). Bar = 2 cm. (**B**) Fresh weight and dry weight of complete plant seedlings under different NaCl treatments. Data are means ± SD of four replicates; different letters indicate significant differences at *p* = 0.05 according to Duncan’s multiple range test.

Given that *Lb1G04202* can improve the salt tolerance of Arabidopsis during seed germination, its effect on salt tolerance at the seedling stage was then explored. We transplanted seedlings into nutrient soil and, after 10 d, watered the seedlings with Hoagland solution with or without 100 mM NaCl. After one week, the leaves of each line grown under the NaCl treatment yellowed, deformed, and wilted to varying degrees (Figure 5A). The NaCl treatment had the strongest effect on the WT; WT plants were smaller and had more serious yellowing and wilting than Col-35S::*Lb1G04202* plants. The biomass data confirmed this trend; transgenic lines had significantly higher fresh and dry weights than the WT under the 100 mM NaCl treatment (Figure 5B).

**Figure 6:**
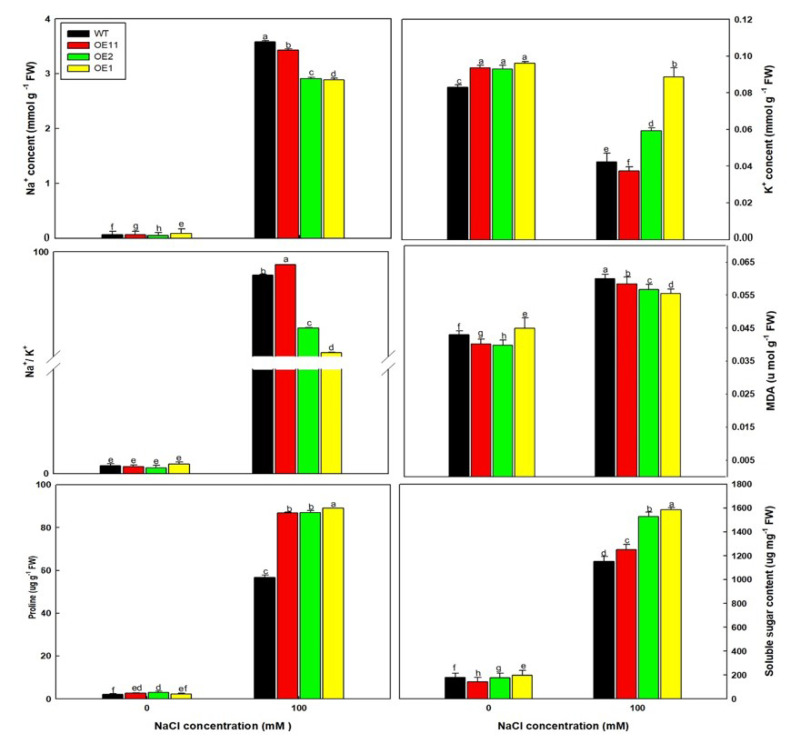
Na^+^, K^+^, MDA, proline and soluble sugar contents of Arabidopsis Col-35S::*Lb1G04202* lines under control and 100 mM NaCl growth conditions. Measurements were performed on 20-d-old seedlings, and four replicates were performed per line. Data are the mean ± SD of four plants; different letters indicate significant differences at *p* = 0.05 according to Duncan’s multiple range test.

Subsequently, various physiological properties of plants under the 0 and 100 mM NaCl treatments were measured (Figure 6). Under 100 mM NaCl, OE lines accumulated more K^+^, proline, and soluble sugar but less Na^+^ and MDA than WT plants. MDA is the product of membrane lipid peroxidation, and therefore it represents the degree of tissue damage in plants [41]. The trend in MDA contents was consistent with that of Na^+^. Therefore, these results suggest that *Lb1G04202* overexpression can reduce the damage caused by NaCl. 

### 2.4. Lb1G04202 Enhances Salt Tolerance by Alleviating Osmotic Stress

**Figure 7:**
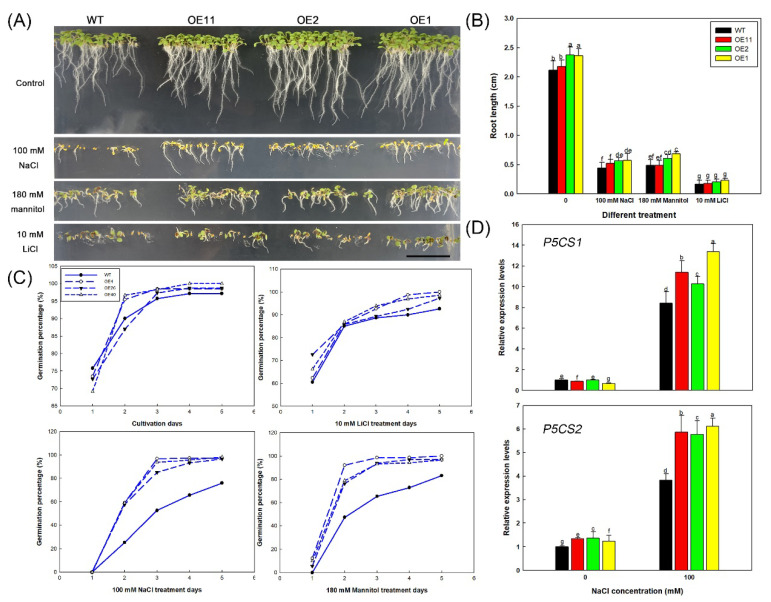
*Lb1G04202* enhances salt tolerance by alleviating osmotic stress in Arabidopsis. (**A**) Col-35S::*Lb1G04202* seeds grown on medium containing 100 mM NaCl, 10 mM LiCl, and 180 mM mannitol for 5 d. Bar = 1 cm. (**B**) Root lengths of 5-d-old seedlings determined using ImageJ software. Thirty seedlings were analyzed per line. Data are the mean ± SD of 30 plants. (**C**) Germination percentage calculated each day after sowing on different media. Forty seeds per line were sown per treatment, and three replicates were performed. Data are the mean ± SD of 40 plants; different letters indicate significant differences at *p* = 0.05 according to Duncan’s multiple range test. (**D**) Relative expression levels of *AtP5CS1* and *AtP5CS2* in Arabidopsis Col-35S::*Lb1G04202* lines. The expression level of each gene was measured by qRT-PCR with three biological replicates (separate experiments); different letters indicate significant differences at *p* = 0.05 according to Duncan’s multiple range test.

NaCl generates osmotic stress and ionic stress [42]. Therefore, to further explore the mechanism by which *Lb1G04202* improves salt tolerance, 180 mM mannitol was used to generate the same osmotic potential as 100 mM NaCl and 10 mM LiCl to generate the same ionic stress as 100 mM NaCl (Figure 7A). Under the isotonic mannitol treatment, the overexpression lines showed the same growth pattern as under the 100 mM NaCl treatment, that is, OE plants had higher germination rates (Figure 7C) and longer roots (Figure 7B) than the WT. Under the 10 mM LiCl treatment, the growth of all lines was severely inhibited, and no growth advantage was detected in the transgenic lines. These results showed that under NaCl treatment, WT plants were subjected to both osmotic and ionic stress, while the transgenic lines of *Lb1G04202* showed higher salt tolerance during germination due to higher resistance to osmotic stress. 

Next, the expression of osmotic response-related genes in the OE lines under 0 and 100 mM NaCl was determined to explore the mechanisms by which *Lb1G04202* improves Arabidopsis salt tolerance. Specifically, we investigated the expression of P5CS1 and P5CS2, which participate in proline synthesis. The expression levels of *AtP5CS1* (*DELTA1-PYRROLINE-5-CARBOXYLATE SYNTHASE1*) and *AtP5CS2* were much higher in Col-35S::*Lb1G04202* lines than those in WT under 100 mM NaCl (Figure 7D). Expression level patterns were consistent with the osmotic stress resistance level and proline accumulation level of these plants. In summary, *Lb1G04202* does enhance genes involved in proline production.

## 3. Discussion

The uncharacterized gene reported here, *Lb1G04202* from recretohalophyte *L. bicolor*, had no conserved domain and no homologous genes among reported species. Due to its position in the salt gland and high expression under salt conditions, we investigated its salt resistance function by heterologously expressing it in Arabidopsis, where it enhanced the salt tolerance of Arabidopsis at the germination and seedling stages by alleviating osmotic stress.

Salt tolerance is a complex trait controlled by multiple mechanisms at the morphological, physiological and molecular levels [43]. For decades, scientists have been devoted to unraveling potential salt tolerance mechanisms, such as the SOS pathway [44,45] and NHX1 unloading transporter [46]. This research has provided the foundation for in-depth investigations of salt resistance. Halophytes have abundant salt-resistance genes and therefore make ideal research material. In particular, recretohalophytes have attracted the attention of researchers due to their specific salt secretory structures and salt excretion function. Among them, *L. bicolor* has a distinct genetic background with a transformation system [29] and mutant library [47]. No reference genome of a species with salt glands has been reported; however, many novel genes with unknown functions have been proposed to participate in salt resistance. 

*Limonium bicolor* is a recretohalophyte with typical salt glands. It can grow normally and complete its life cycle under high levels of NaCl [6,8]. Salt glands are typical epidermal structures that secrete salt. Many genes have been proposed to participate in salt resistance and salt gland development [26,32,33] in *L. bicolor*, such as *LbTTG1*, *LbTRY* and *LbSAD2* [30,31,32]. However, these genes are all homologs of known functional genes. In other halophytes, functional verification is always carried out in genes with clear domains, for instance, the transformation of Glycerol-3-Phosphate Acyltransferase from *Suaeda salsa* improves salt tolerance in Arabidopsis [48] and the overexpression of genes related to glycine betaine biosynthesis from *Salicornia europaea* improves salt-tolerance of tobacco [49]. For *L. bicolor*, which has salt glands, the unknown functional genes, such as *LbHLH*, may play a significant role in salt resistance [33]. We believe that the unknown functional genes in *L. bicolor* may be unique to salt gland development. In fact, uncharacterized functional genes have rarely been studied due to unclear domains [50]. Recent studies have identified many novel genes involved in plant growth and morphological construction, for example, *PebHLH35* from *Populus euphratica* improves drought resistance when overexpressed in Arabidopsis [36] and a WD40-repeat protein from the halophyte *C.* *quinoa* is related to the formation of epidermal bladder cells [37]. Therefore, identifying the function of unknown genes expressed in *L. bicolor* may be important for understanding salt gland development and salt resistance. 

In the current transformation of *Lb1G04202* in Arabidopsis, the transgenic lines showed enhanced salt tolerance in biomass and germination with increasing exogenous expression, and a typical dose effect could be observed. There may be two main reasons for the salt tolerance. On the one hand, with respect to the physiological mechanism, transgenic lines overexpressing *Lb1G04202* accumulated less Na^+^ than the WT, which may be related to the increased absorption of K^+^, proline and soluble sugar. This is consistent with previous reports that salt-tolerant lines often have low Na^+^ content to avoid salt damage and a high accumulation of K^+^ instead [51,52]. In particular, the high expression level line was the most tolerant to salt, indicating that salt tolerance was proportional to gene expression level. 

On the other hand, in general, NaCl results in both osmotic stress and ionic stress [42]. Therefore, to untangle the effect of *Lb1G04202* on NaCl tolerance, we simulated osmotic stress and ion stress, separately. Iso-osmotic mannitol was applied to generate similar osmotic stress of NaCl. The transgenic lines showed high resistance to osmotic stress, similar to their response to NaCl treatment. This indicates that expression of *Lb1G04202* in Arabidopsis can directly resolve the osmotic stress generated by NaCl. Moreover, the expression of *P5CS1* and *P5CS2*, which encode key rate-limiting enzymes in proline synthesis and thus affect osmoregulation [53], were higher in OE lines that in the WT. This corresponded with the high proline contents in the Col-35S::*Lb1G04202* lines. Proline is an important organic osmotic substance [54,55,56] that can reduce osmotic potential and prevent plant water loss, alleviating osmotic stress. While transgenic plants showed tolerance to osmotic stress, they were not tolerant to LiCl (Figure 7). This indicates that the enhanced tolerance of transgenic lines to NaCl stress was due to the enhanced tolerance to osmotic stress and not ion stress.

In our study, *Lb1G04202* was localized to the nucleus. Therefore, it may regulate gene expression via direct or indirect binding to promoters of target genes, which will be further investigated in future studies. Moreover, many novel genes have been reported halophytes such as *L. bicolor*. These genes could be further explored to develop salt-resistant crops and improve saline land.

## 4. Materials and Methods

### 4.1. Plant Materials and Growth Conditions

The seeds of wild *Limonium bicolor* were obtained from a site with saline soil (N37°40′; E118°55′) in the Yellow River Delta (Shandong Province, China). The completely dried seeds were stored at 4 °C for at least six months before use. The dried seeds were sterilized with 70% ethanol for 5 min and shaken with 6% (*v*/*v*) NaClO (sigma, City of Saint Louis, MO, USA) for 18 min. Then, the seeds were washed with sterile distilled water 5–7 times and soaked for another 20 min. After disinfection, the seeds were germinated on Murashige and Skoog (MS) (Zkorigin Bioscience Co., Ltd., Beijing, China) basic medium with 0.9% agar in Sterile Petri dish to obtain sterile seedlings. Seedlings were then cultured in the flowerpots (10 cm × 10 cm) with nutrient soil (soil: vermiculite: perlite, 3:1:1) at 28 ± 3 °C/23 ± 3 °C (day/night), 600 µmol/m^2^/s (15 h photoperiod) photon flux density and 70% relative humidity.

*Arabidopsis thaliana* ecotype Col-0 (Columbia-0) seeds were disinfected by shaking with 70% ethanol twice (5 min each time) and 95% ethanol three times (4 min each time). After 2 d of vernalization at 4 °C, the seedlings were cultivated at 22 °C/18 °C (day/night) under a 16 h/8 h photoperiod with a photon flux density of 150 µmol/m^2^/s and 70% relative humidity. The seedlings were cultured on 1/2 MS medium for one week and transplanted into pots (10 cm height × 10 cm diameter) filled with nutrient soil (soil: vermiculite: perlite, 3:1:1). After the inflorescence had grown, plants were infected and transformed using *Agrobacterium tumefaciens*.

### 4.2. Cloning and Bioinformatic Analysis of Lb1G04202

To extract the total RNA of *L. bicolor*, the first true leaves of *L. bicolor* were collected at the following developmental stages: the undifferentiated stage (stage A; ~5000 leaves), salt gland differentiation stage (stage B; ~4000 leaves), stomata differentiation stage (stage C; ~3000 leaves), epidermis differentiation stage (stage D; ~1000 leaves) and mature stage (stage E; ~1000 leaves) [40]. Total RNA was extracted from leaves using a FastPure Plant Total RNA Isolation kit (RC401-01; Vazyme Biotech Co., Ltd., Nanjing, China). cDNA was reverse transcribed from the RNA with 2× T5 Fast qPCR Mix (SYBR Green I) (Tsingke Biological Technology, Beijing, China) according to the manufacturer’s instructions.

The full-length sequence of *Lb1G04202* was cloned with primers *Lb1G04202*-S and *Lb1G04202*-A (Table S1), which were designed with Primer Premier 5.0. The cDNA of *L. bicolor* was used as the template. The online tool EXPASY (http://www.bio-soft.net/sms/index.html, accessed on 8 April 2022) was used to translate the protein encoded by the gene. The structure and conserved domain of Lb1G04202 were predicted using the online tool SMART (http://smart.embl-heidelberg.de/, accessed on 8 April 2022). The coding protein sequences were applied in NCBI-BLAST (https://blast.ncbi.nlm.nih.gov/Blast.cgi, accessed on 8 April 2022) to analyze sequence similarity.

### 4.3. Localization of Lb1G04202

The circular pCAMBIA1300 plasmid was digested by SalI to obtain a linear pCAMBIA1300 vector. The *Lb1G04202* fragment carrying the SalI digestion site was amplified with the primers *Lb1G04202*-OE1-S and *Lb1G04202*-OE1-A (Table S1). Homologous recombination was used to connect the *Lb1G04202* fragment with the SalI digestion site to the linear pCAMBIA1300 vector to form a recombinant circular expression vector pCAMBIA1300-*Lb1G04202*. This was done with a ClonExpress II One Step Cloning Kit (Vazyme Biotech Co., Ltd., China) according to the manufacturer’s instructions. After transforming *Agrobacterium tumefaciens* GV3101, the recombinant expression vector pCAMBIA1300-*Lb1G04202* was transformed into onion epidermal cells to explore the subcellular localization of *Lb1G04202*. After 2 d of cultivation in the light, fluorescent signals of GFP-labeled *Lb1G04202* were detected under a TCS S8 MP two-photon laser-scanning confocal microscope (Leica, Heidelberg, Baden-Württemberg, Germany). DAPI was used to locate the nucleus specifically and was observed under excitation at 358 nm. FM4−64 (N-(3-triethylammoniumpropyl)-4-(6-(4-(diethylamino)phenyl) hexatrienyl) pyridinium dibromide)(Invitrogen, Carlsbad, CA, USA) was used to locate the plasma membrane and the signal was observed under excitation at 559 nm.

To determine the expression position of *Lb1G04202* in *L. bicolor*, developing leaves (the first true leaf at 6–8 d after germination) were isolated from *L. bicolor* for in situ hybridization. Briefly, the leaves were fixed in 4% paraformaldehyde, embedded in paraffin, and dehydrated through an alcohol series. Thin sections (8 µm) of tissue were treated with proteinase K and hybridized in 6 ng/µL hybridization solution at 37 °C overnight. The digoxin-labeled *Lb1G04202* probe (5′-DIG-GCCCUAUACAUCCUUUCAGCACCAUCUUCAU-3′, purified by HPLC) appeared blue-violet. The negative control used the sense strand labeled with digoxin.

### 4.4. Analysis of the Expression Pattern of Lb1G04202 during the Development of L. bicolor and under Different Treatments

*Limonium bicolor* was cultivated on MS medium containing different additives (100 mM NaCl, 0.04 mg/L 6-BA and 0.1 mg/L abscisic acid) and the seedlings were collected for RNA extraction. In addition, the A–E stage leaves, stems, roots and mature leaves of *L. bicolor* grown on MS medium were harvested; these materials were used for RNA extraction individually. 

Plant total RNA was reverse transcribed to obtain cDNA, and quantitative RT-PCR was performed in a 20-μL reaction system containing 10 μL SYBR qPCR Master Mix (Vazyme Biotech Co., Ltd.), 0.2 µM primers (Table S1), and 300 ng cDNA. The PCR was conducted in a fluorometric thermal cycler (Bio-Rad CFX96 Realtime PCR System) with the following conditions: pre-incubation at 94 °C for 30 s; 40 cycles of denaturation and annealing at 94 °C for 5 s and 60 °C for 30 s; and melting at 95 °C for 15 s, 60 °C for 60 s, and 95 °C for 1 s. *Lbtubulin* was used as an internal control [30,31,32,33,57]. The *Lbtubulin*-RT-S, *Lbtubulin*-RT-A, *Lb1G04202*-RT-S and *Lb1G04202*-RT-A primers (Table S1) were designed using Beacon Designer software (version 7.8). The expression level of *Lb1G04202* in different tissues was calculated relative to the expression level in the stem (which was set to 1). Three biological replicates (separate experiments) were performed. Relative expression levels were calculated using 2^–ΔΔC(T)^.

### 4.5. Heterologous Expression in Arabidopsis Lines

The circular pCAMBIA3301 vector was digested by NcoI to obtain a linear pCAMBIA3301 vector. To connect the *Lb1G04202* fragment with the linear pCAMBIA3301 vector, the full-length coding sequence (CDS) of *Lb1G04202* carrying NcoI digestion sites was amplified with the primers *Lb1G04202*-OE2-S and *Lb1G04202*-OE2-A (Table S1). Plasmid p35S::*Lb1G04202* was generated by homologous recombination using a ClonExpress II One Step Cloning Kit (Vazyme Biotech Co., Ltd., Nanjing, China) to express *Lb1G04202* under the control of the CaMV 35S promoter. p35S::*Lb1G04202* was transformed into *Agrobacterium tumefaciens* GV3101 cells for heterologous overexpression before transforming the Arabidopsis by floral dip. Harvested Arabidopsis seeds were cultured in nutrient soil. After three generations of screening with Basta (0.1%, *v*/*v*), the Arabidopsis seeds homozygous for the p35S::*Lb1G04202* transgene were obtained.

The DNA extracted from the leaves of transgenic lines was used as a template, and the primers pCAMBIA3301-S and pCAMBIA3301-A (Table S1) were used to amplify *Lb1G04202*. Agarose gel electrophoresis was used to identify the positive transgenic plants. Then, mRNA was extracted from different Col-35S::*Lb1G04202* lines using a FastPure Plant Total RNA Isolation kit (Vazyme Biotech Co., Ltd.) according to the manufacturer’s instructions. *Lb1G04202* expression levels in different Col-35S::*Lb1G04202* lines were analyzed by qRT-PCR using the primers *Lb1G04202*-RT-S and *Lb1G04202*-RT-A (Table S1). *AtACTIN* (primers *Atactin*- RT-S and *AtACTIN*- RT-A, Table S1) of Arabidopsis was used as the internal control. Since the *Lb1G04202* gene of *L. bicolor* has no homologous gene in Arabidopsis, the line with the lowest *Lb1G04202* expression level (OE11) was used as the control (the relative expression level was set to 1) to calculate the expression level of *Lb1G04202* in other Col-35S::*Lb1G04202* lines. Three biological replicates were performed for each group. Three Col-35S::*Lb1G04202* lines were selected for physiological characterization based on *Lb1G04202* expression: lines with high (OE1), medium (OE2), and low expression (OE11) level.

### 4.6. Determination of Salt Tolerance Index during Germination

OE11, OE2, OE1 and wild-type (WT) Arabidopsis seeds were cultured on 1/2 MS medium (1% agar) containing different concentrations of NaCl (0, 50, 100, and 150 mM) to analyze the salt tolerance of Col-35S::*Lb1G04202* overexpression lines. We conducted three repeated experiments. Each repeated test contained 40 seeds per line. The number of germinated seeds was counted every day for the first 1–5 d. Specifically, seeds with a radicle >1 mm long that had emerged from the seed coat were considered to be germinated. The emergence of green cotyledons was used as an indicator of cotyledon growth. The cotyledon growth percentage of each line was measured after 3 d of germination. Cotyledon growth percentage (%) = (number of seeds with cotyledons/number of all tested seeds) × 100%. After continuous cultivation for 5 d on different media, the root lengths of different lines were measured using ImageJ software. At least 30 seeds were used for repeated experiments for each index measurement.

### 4.7. Measurement of Physiological Indexes under Salt Treatment at Seedling Stages

The seedlings (OE11, OE2, OE1, and WT) grown on 1/2 MS basic medium for 5 d were separately transplanted into nutrient soil. After two weeks of adaptation to growth, they were irrigated with different concentrations of NaCl (0, 50, 100, and 150 mM NaCl dissolved in Hoagland solution, pH 6.2). Leaf tissue (0.5 g fresh weight per replicate) was harvested from seedlings under the 0 or 100 mM NaCl condition to measure physiological indicators. The Na^+^, K^+^, proline, malonyldialdehyde (MDA), and soluble sugar contents were measured as described previously [30,31,32,33,57]. Ion concentrations were measured with a flame photometer (Cole-Parmer, Chicago, IL, USA). Four replicates per measurement were performed for each line.

### 4.8. Performance of Transgenic Lines under Single Ion and Osmotic Stress

Four Arabidopsis lines (OE11, OE2, OE1, and WT) were cultured in 1/2 MS medium containing 10 mM LiCl (the same ionic effect as 100 mM NaCl) and 1/2 MS medium containing 180 mM mannitol (the same osmotic pressure as 100 mM NaCl) for 5 d. To determine salt tolerance, the germination percentage (%), cotyledon growth percentage (%) and root length were measured with the same methods as those mentioned above. The growth of seedlings under either ion or osmotic stress was compared among different lines to determine why *Lb1G04202* alleviates salt stress and improves salt tolerance.

### 4.9. Expression Analysis of Osmotic Stress-Related Marker Genes in Transgenic Arabidopsis

Four Arabidopsis lines (OE11, OE2, OE1, and WT) were cultured in 1/2 MS medium containing 0 or 100 mM NaCl for 10 d. The RNA of eight samples was extracted with a FastPure Plant Total RNA Isolation kit (RC401-01; Vazyme Biotech Co., Ltd., Nanjing, China). Products obtained after RNA reverse transcription were used for qRT-PCR to analyze the expression of related marker genes under salt stress, including *DELTA1-PYRROLINE-5-CARBOXYLATE SYNTHASE 1* (*AtP5CS1*) and *AtP5CS2* (Table S1). Three biological replicates were performed. *AtACTIN* was used as the internal control.

### 4.10. Statistical Analysis

Statistical significance at *p* = 0.05 (Duncan’s multiple range tests) was determined using SPSS. ANOVA with orthogonal contrasts and mean comparison procedures was used to detect significant differences among treatments.

## 5. Conclusions

We determined that the uncharacterized *Lb1G04202* of *L. bicolor* is localized in salt glands and expressed in the nucleus. Overexpression of *Lb1G04202* in Arabidopsis alleviated osmotic stress by enhancing the expression of *AtP5CS1* and *AtP5CS2* to produce more proline under salt stress. These results indicate that *Lb1G04202* confers salt tolerance by directly affecting the osmotic stress tolerance of plants and could therefore be useful for breeding salt-tolerant crops.

## Data Availability

The datasets used and/or analyzed during the current study are available from the corresponding author on reasonable request.

## Supplementary Materials

The following supporting information can be downloaded at: https://www.mdpi.com/article/10.3390/ijms23105401/s1.

## Abbreviations

*L. bicolor*	*Limonium bicolor*
TTG1	TRANSPARENT TESTA GLABRA1
SAD2	SUPER SENSITIVE TO ABA AND DROUGHT2
TRY	TRIPTYCHON
CPC	CAPRICE
AtSOS1	SALT OVERLY SENSITIVE 1
AtSOS3	SALT OVERLY SENSITIVE 3
AtP5CS1	DELTA1-PYRROLINE-5-CARBOXYLATE SYNTHASE 1
AtP5CS2	DELTA1-PYRROLINE-5-CARBOXYLATE SYNTHASE 2
Col-0	Columbia-0
qPCR	quantitative PCR
CDS	coding sequence
WT	wild-type
