# Supplementary material for: Lb1G04202, an Uncharacterized Protein from Recretohalophyte Limonium bicolor, Is Important in Salt Tolerance
Source: Int J Mol Sci. 2022 May 12;23(10):5401. doi: 10.3390/ijms23105401 (PMC9140551; doi:10.3390/ijms23105401)
Supplement: Supplementary file 1 [file ijms-23-05401-s001.zip › ijms-1716486-supplementary.pdf]

Table S1 The primers used in this study

Name	Oligonucleotide sequence	Role
Lb1G04202-S	5-ATGACCGAAGAGGAGAAAG -3	Full length amplification
Lb1G04202-A	5- TCCTTCGATTCAGCTATGG-3	
Lb1G04202-OE1-S	5- CGGGGATCCTCTAGAGTCGACATGACCGAAGAGGAGAAAGCC-3	Construction of p35S:: *Lb1G04202*-GFP vectors digested with Sal Ⅰ
Lb1G04202-OE1-A	5-GCCCTTGCTCACCATGTCGACTCAATCCTTCGATTCAGCTATGG -3	
Lb1G04202-RT-S	5-CAAGGTGAATCTAAAGTTCTA -3	Real-time PCR in different developmental stages, condition and Arabidopsis overexpression lines
Lb1G04202-RT-A	5- GCTCTTTAATAAACATCATCTC-3	
Lbtubulin-RT-S	5-GGTTGAGTGAGCAGTTCAC-3	
Lbtubulin-RT-A	5-GATAACCAGCCACACCTTAGC-3	
Atactin- RT-S	5-GGTAACATTGTGCTCAGTGGTGG-3	
Atactin- RT-A	5-AACGACCTTAATCTTCATGCTGC-3	

Lb1G04202-OE2-S	5-ACGGGGGACTCTTGACCATGGATGACCGAAGAGGAGAAAGCC -3	
Lb1G04202-OE2-A	5-TTACCCTCAGATCTACCATATCCTTCGATTCAGCTATGGTTG -3	Construction of Col-35S:: *Lb1G04202*
pCAMBIA3301-S	5-GGATTCCATTGCCCAGCTATCT -3	
pCAMBIA3301-A	5-GGTTTCTACAGGACGGACGAGT -3	
AtP5CS1- RT-S	5-CAAGATGAGATTACATTCG-3	
AtP5CS1- RT-A	5-GGTTATGATGACAGGAAT-3	RT-qPCR verification of osmotic response related marker genes in Col-35S:: *Lb1G04202*
AtP5CS2- RT-S	5-GTGACGGAAGATAGTGAA-3	
AtP5CS2- RT-A	5-TCCTGCTTGTGCTTATTC-3	
